# Sex Determination Using Human Sphenoid Sinus in a Northeast Iranian Population: A Discriminant Function Analysis

**DOI:** 10.30476/dentjods.2022.92915.1685

**Published:** 2023-03

**Authors:** Seyed Ahmad Banihashem Rad, Najmeh Anbiaee, Shahin Moeini, Ali Bagherpour

**Affiliations:** 1 Graduate School for Health Sciences, Dept. of Restorative, Preventive and Pediatric Dentistry, School of Dental Medicine (ZMK Bern), University of Bern, Switzerland; 2 Oral and Maxillofacial Diseases Research Center, Mashhad University of Medical Sciences, Mashhad, Iran; 3 Postgraduate Student, Dept. of Oral and Maxillofacial Radiology, School of Dentistry, Mashhad University of Medical Sciences, Mashhad, Iran; 4 Dept. of Oral and Maxillofacial Radiology, School of Dentistry, Mashhad University of Medical Sciences, Mashhad, Iran

**Keywords:** Forensic Anthropology, Sex Determination by Skeleton, Cone-Beam Computed Tomography, Discriminant Analysis, Iran

## Abstract

**Statement of the Problem:**

Sex determination, using skeletal remains, is of paramount importance in forensic studies. The skull accounts for the most sexual dimorphism after the pelvis. Recent studies have shown that paranasal sinuses are valuable in sex determination and considering the location of the sphenoid sinus, the risk of traumatic injuries to this structure is low.

**Purpose:**

The present study aimed to evaluate the morphology of the sphenoid sinus and determine the validity of sphenoid sinus volume (SSV) in sex determination using cone beam computed tomography (CBCT) images.

**Materials and Method:**

In this cross-sectional retrospective study, CBCT images of 469 Iranian patients (186 male and 283 female), aged 24-45 years, were selected. The morphology of the sphenoid sinus was recorded. 3D Slicer software (4.10.0) was used to assess SSVs in coronal and axial planes. For data analysis, t-test, chi-square test, and discriminant function analysis (DFA) were performed using predictive analytics software (ver. 18.0).

**Results:**

The most common morphology of the sphenoid sinus in both genders was the sellar type (50.5%). SSV was significantly larger in males than in females (*p*< 0.001). DFA showed that the capability of SSV in sex identification was 86.0% and 92.9% in males and females, respectively.

**Conclusion:**

The findings of this study suggest that SSV is a reliable variable in gender discrimination in a northeast Iranian population. However, since the morphology of the sphenoid sinus and sex were independent of each other, the morphology of the sphenoid sinus is not a suitable indicator for sex determination.

## Introduction

Numerous studies have shown that human remains play a key role in sex determination, including fingerprint, dental morphology, cheiloscopy, and radiologic examination. However, only hard tissues, such as bones, of human remains remain intact over time [ 1 - 2 ].

Sex determination from human bones is usually performed using metric and morphological (non-metric) analyses. Metric analyses are quantitative and objective and use anatomical references; therefore, they are repeatable [ 3 ]. On the contrary, morphologic analyses are qualitative and subjective, and therefore observing the morphology as well as the experience of the observer plays a fundamental role, which is a limitation [ 3 - 4 ].

Bones exhibiting the most marked sexual dimorphism include the pelvis, cranium, and long bones [ 5 ]. In cases where the skeleton is well-preserved, sex determination is 100% accurate. However, bones, particularly the pelvis and long bones, are usually found in separate pieces, thus often making sex determination difficult [ 6 ]. Given that only the cranium is preserved in most severe events, sex determination studies using the dimension and morphology of the cranium have significantly increased [ 7 ]. Paranasal sinuses are cranial components consisting of frontal, ethmoidal, maxillary, and sphenoid sinuses. These structures remain intact in most cases; therefore, they can be used in sex determination according to their dimensional characteristics [ 6 , 8 - 9 ].

The sphenoid sinuses are paired spaces formed within the body of the sphenoid bone beneath the sella turcica. Due to their location inside the skull, these sinuses are at low risk for traumatic and pathologic injuries. Furthermore, sphenoid sinuses develop earlier than other paranasal sinuses and reach their adult size at around 12 years of age [ 10 ]. Depending on the extent to which the sphenoid bone is pneumatized, the sphenoid sinus is categorized into conchal, presellar, sellar, and postsellar types [ 11 - 12 ]. A detailed preoperative radiomorphometric assessment of the sphenoid sinus is necessary to ensure a successful transsphenoidal endoscopic surgery [ 13 - 14 ]. This is because the sinus is surrounded by vital structures including the internal carotid artery, optic nerve, and maxillary nerve [ 15 ]. From a forensic point of view, if the sphenoid sinus morphology and gender are not independent of each other, the finding could be useful in distinguishing between the sexes.

There are various methods for morphometric and volumetric assessment of the sphenoid sinus; however, cone beam computed tomography (CBCT) is preferred over other methods such as computerized tomography (CT) due to low X-ray exposure, minimum costs, and field of view (FOV) compatibility with the understudy region [ 16 - 17 ]. Given that the skeletal properties of individuals differ in various populations due to environmental, nutritional, and genetic factors, it is necessary to form specialized models of sex determination to generate reliable and accurate information [ 18 ].

The present study aimed to evaluate the relationship between the morphology of sphenoid sinus and sex and to compare the sphenoid sinus volume (SSV) between the two genders. In addition, the main purpose of our research was to construct discriminant function models for sex determination from the sphenoid sinus in the northeast Iranian population.

## Materials and Method

### Sample selection

In this retrospective study, 469 CBCT images of 564 patients who were referred to a private oral and maxillofacial radiology clinic in Mashhad, Iran, were studied (Table 1). The patients were referred for paranasal sinus examination between 2017 and 2021. All patients had normal dentition. Patients with a history of trauma, head and neck syndromes, and increased mucosal thickness in each of the paranasal sinuses were excluded from the study (95 patients).

**Table 1:** Descriptive findings of the patients’ age

	Males	Females	Total
Number of samples (%)	186 (39.7)	283 (60.3)	469 (100)
Minimum	25	24	24
Maximum	45	45	45
Mean±SD.	35.06±5.27	34.17±4.73	34.52±4.97
95% CI	34.30; 35.82	33.62; 34.73	34.07; 34.98

CI: confidence interval; S.D.: standard deviation

All the CBCT images were prepared using Promax 3D Mid (Planmeca Oy, Helsinki, Finland) unit. Exposure factors were as follows: 90 kVp, 4 mA, acquisition time 13.5 s, voxel size 0.4 mm, and FOV 16.0×17.5cm.

### Volumetric and morphological assessment

**Figure 1:**
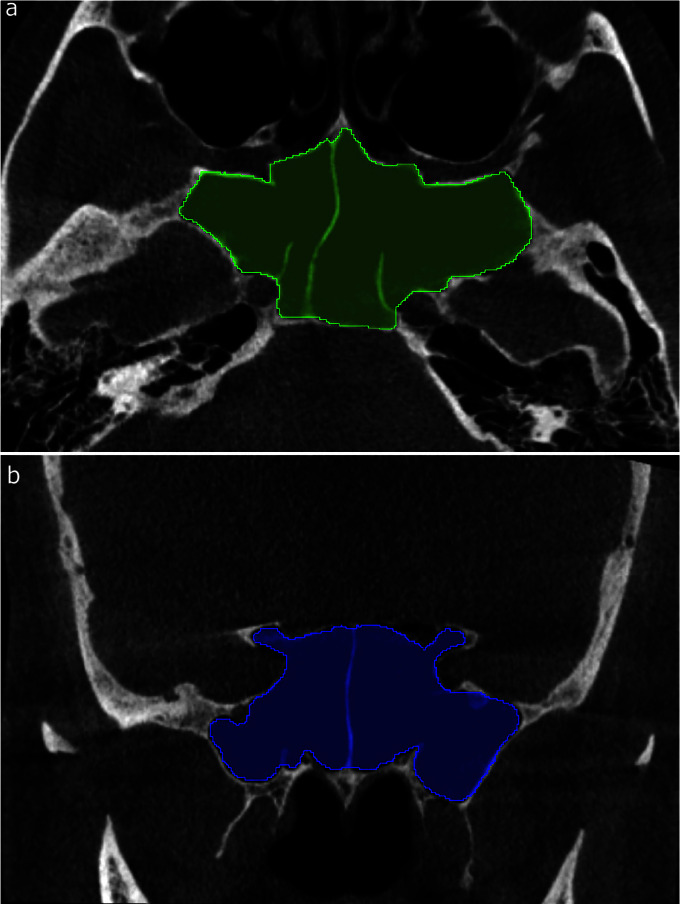
Sphenoid sinus segmentation using 3D Slicer software. **a:** Axial (green) and **b:** coronal (blue) planes

**Figure 2:**
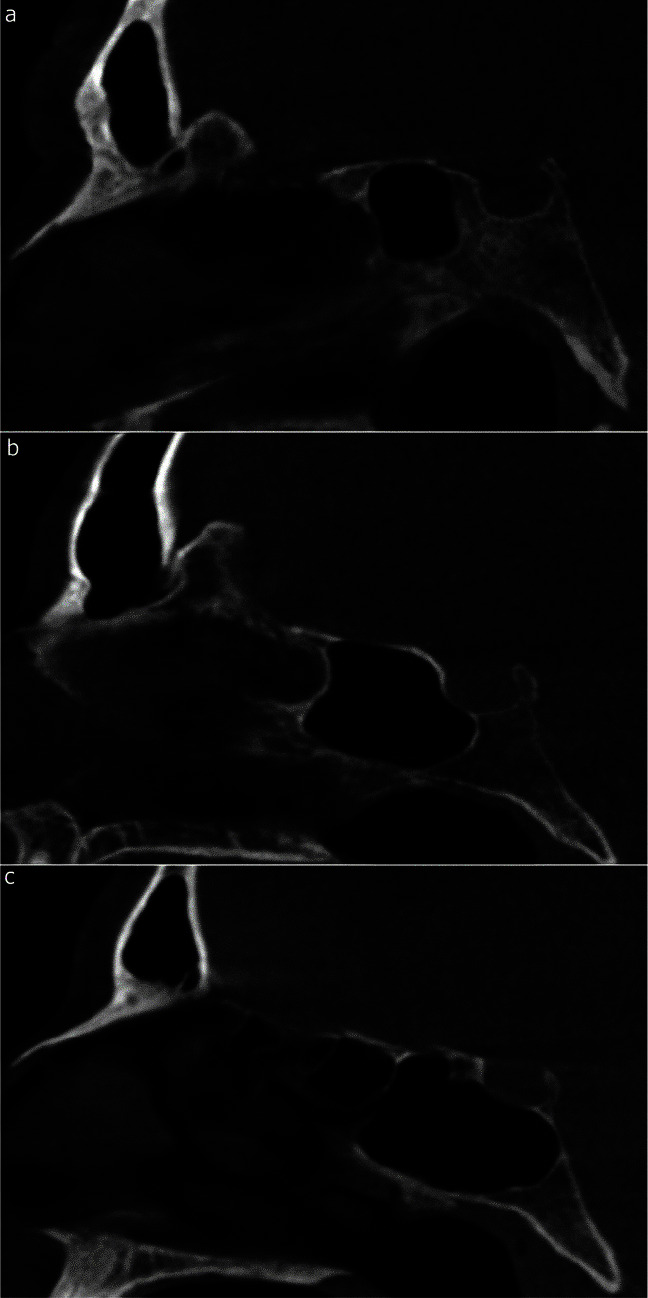
Different types of sphenoid sinus pneumatization. Midsagittal cone beam computed tomography images of sphenoidal sinus morphology; **a:** Presellar (posterior wall of the sphenoid sinus extends posteriorly in front of the anterior wall of the sella turcica); **b:** Sellar (posterior wall of the sphenoid sinus lies between the anterior and posterior walls of the sella turcica); and c: Postsellar (posterior wall of the sphenoid sinus is behind the posterior wall of the sella turcica). No conchal types (placed as small spaces in front of the anterior wall of the sella turcica) were observed in our study

3D Slicer (version 4.10.0; https://www.slicer.org) was used for the assessment of SSV. First, a Digital Imaging and Communications in Medicine (DICOM) format of the file was imported into the 3D Slicer, then the area was manually painted alternately (one out of two slices) in the segment editor using the paint tool [ 19 ]. Using the Fill between slices option, the unpainted slices were painted via the interpolation process; ultimately, the segmentation and volume measurement processes were completed in the Data and Models modules [ 20 - 21 ]. It should be noted that the segmentation and volume determination were conducted in the two axial and coronal planes (Figure 1a, b), and the mean of the two was considered as the SSV. The images were observed and the volumes were accordingly measured using a 22-inch LCD monitor with a 1920×1080-pixel screen resolution. Morphological classification of the sphenoidal sinus (Figure 2a-c) and volume measurements were conducted by an oral and maxillofacial radiologist with around 15 years of experience (A.B.). The intra-observer error for SSV measurements was calculated after a 1-month interval by re-measuring the SSV in 50 (≈10%) samples. The two measurements were then analyzed using paired t-test.

### Statistical analysis

Predictive analytics software version 18.0 (SPSS Inc., Chicago, IL, USA) was used for statistical analysis. The Kolmogorov-Smirnov (K-S) test was used to evaluate the distribution of age and SSV. SSV comparison between the two genders was conducted using the independent t-test, and evaluation of the independence of the study’s qualitative variables (gender and sinus morphology) was conducted using the chi-square test. Ultimately, discriminant function analysis (DFA) was used to determine if sinus volume could be used in sex differentiation. If the p value was less than 0.05, the results were considered statistically significant.

## Results

The assessment of normality distribution of age and SSV was conducted in all samples for each gender using the K-S test. The K-S test showed that age and volume data were normally distributed (*p*> 0.05). Therefore, parametric tests were used in all analyses. In addition, we tested the intra-observer error to verify the reliability of the SSV measurements. Evaluation of the SSV using paired t-test in a 1-month interval showed no significant difference (*p*= 0.68). Moreover, the evaluation of mean age in the two understudied genders using an independent t-test did not indicate any significant difference (*p*=0.07).

### Morphological analysis

The conchal type morphology was not observed in any of the samples in this study. The relationship between sphenoid sinus and gender is shown in Table 2. Pearson’s chi-square test showed that the morphology of sphenoid sinus and gender were independent of each other (χ^2^= 0.93, *p*= 0.63). The most common morphology in the two genders was the sellar type (around 50% of the cases) (Table 2).

**Table 2:** Cross tabulation of gender and sphenoid sinus morphology

		Gender		Total
		Male (%)	Female (%)	
Sphenoid	Presellar type	20(10.8)	23(8.1)	43(9.2)
Sinus	Sellar type	92(49.5)	145(51.2)	237(50.5)
Morphology	Postsellar type	74(39.8)	115(40.6)	189(40.3)
Total		186(100.0)	283(100.0)	469(100.0)

Pearson Chi-Square (χ^2^)= 0.93, *p*= 0.63

### Volumetric dimension analysis

The independent t-test showed a significant difference in SSV between the two genders (*p*< 0.001). The SSV in sellar and postsellar types was significantly different between the two genders (*p*< 0.001). However, this finding was not observed in the presellar type (*p*= 0.19) (Table 3). 

**Table 3:** Mean (±S.D.) of volumetric measurements (cm^3^) of the sphenoid sinus in both genders

	Volumetric Measurements (cm^3^±SD)			
	Unclassified	Presellar	Sellar	Postsellar
Male	14.12±4.11	5.98±1.50	14.52±2.88	15.83±3.26
Female	7.48±2.33	5.37±1.52	6.34±1.36	9.34±2.16
*p*	<0.001	0.19	<0.001	<0.001

Unclassified: The morphology of the sphenoid sinus was not considered; S.D.: standard deviation

### Discriminant function analysis (DFA)

Considering the morphology of the sphenoid sinus and gender were independent of each other, the morphology of the sphenoid sinus was not used in DFA for sex determination. In the present study, SSV is an independent variable in sex determination. According to Table 4, the discriminant formula is as follows:

Discriminant Score = SSV × 0.316 + (-3.200)

**Table 4:** Discriminant function analysis using the sphenoid sinus volume (SSV) to discriminate between males and females

Canonical Discriminant Function Coefficients					
		SSV	Constant		
Unstandardized coefficient		0.316	-3.200		
Discriminant Score (D)= SSV×0.316+(-3.200)					
Functions at group centroids		Female	Male		Classified as male if D >
		-0.833	1.268		0.217
			Predicted Group Membership		
Gender			Male	Female	Total
Original	Count (%)	Male	160(86.0)	26(14.0)	186(100)
		Female	20(7.1)	263(92.9)	283(100)
90.2% of original grouped cases correctly classified.					

Group centroids (Table 4) indicate the mean discriminant scores for each gender. The sectioning point (0.217) was calculated in mean group centroids for females and males. Using the formula above, if the discriminant score is greater than the sectioning point, the sample is classified as male, and if it is lower than the sectioning point, it is classified as female. It should be noted that the more distant the discriminant score is from the sectioning point, the more accurate is the sex determination.

The sex classification accuracy of the DFA is shown in Table 4. In general, 86.0% (160/186) of the males and 92.9% (263/283) of the females were classified correctly. Misclassification error was reported more in males (14.0%) than in females (7.1%). This difference could be attributed to the higher number of females as compared to males in the present study. The overall accuracy of the DFA was 90.2%.

## Discussion

Sex determination is an important aspect of forensic medicine. Various studies on sexual dimorphism in human bones require the establishment of specialized anthropometric standards for different populations [ 5 , 22 ]. Therefore, this study was conducted due to the lack of similar studies on the morphology and SSV in sex determination in the Iranian population.

The sphenoid sinuses are a pair of air-filled spaces in the sphenoid bone. These cavities are small at birth, and their dominant growth takes place after puberty. They expand posteriorly into the presellar region in infancy, and then extend into the region under and behind the sella turcica, eventually reaching full size throughout adolescence [ 23 ]. Furthermore, their deep location inside the skull makes them less vulnerable to injuries and pathologic changes, thus remaining intact on a dry skull [ 10 ].

Various studies have been conducted in forensic medicine using CBCT devices [ 9 , 15 , 18 , 24 - 26 ]. Compared to multislice CT, CBCT has great advantages of lower imaging costs, ease of use, and higher resolution in particular [ 27 ]. Various studies [ 24 , 28 ] have suggested the application of CBCT images in the evaluation of paranasal sinuses. Dimensional and volumetric accuracy in CBCT is superior to conventional CT. Since CBCT has isotropic voxels in all directions, except for the high resolution of axial planes in CT, longitudinal planes are reformatted by a summation of axial CT images [ 29 ].

In this study, a 3D Slicer was used to measure the volume of the sphenoid sinus. 3D Slicer is a free, open source, and multi-platform medical image processing software. This software works both manually and semi-automatic for segmentation. Furthermore, it is DICOM compatible and does not require any advanced hardware, which is one of its added advantages [ 19 ]. According to numerous studies [ 20 - 21 , 30 ], 3D slicer has a high level of accuracy in segmentation and volume rendering, thus making it an appropriate choice for the current research.

Some studies have found a relationship between dentition status and changes in the skull components [ 31 - 32 ]. This relationship supports the biomechanical theory suggesting the distribution of masticatory forces from the mandible to the cranium. Since the sphenoid sinus is located at the base of the sphenoid bone, changes in the shape and size of this bone are associated with cranial growth [ 33 ]. Therefore, similar to Ramos *et al*.’s study [ 25 ], patients with complete dentition were selected to avoid possible changes in the measurements in the present study. Furthermore, the absence of pathologic changes in paranasal sinuses and the adjacent tissues was an important factor considered in this study.

Reliability and reproducibility of measurements are of paramount importance in forensics; therefore, the measurements should be made by experts who have a comprehensive understanding of the studied subjects. All the measurements were conducted by an oral and maxillofacial radiologist (A.B.) with around 15 years of experience. The paired t-test showed no significant difference in the before and after volume measurements (*p*= 0.68), showing an acceptable intra-observer agreement in the present study.

Linear analyses are not sufficiently accurate due to the anatomic complexities of the paranasal sinuses [ 34 ]. A study conducted by Ramos *et al*. [ 25 ] using CBCT images measuring linear and volumetric dimensions of the sphenoid sinus in 268 Brazilian patients concluded that linear measurements were not significantly different between the two genders (*p*> 0.05); however, SSV significantly differed (males 11.36±4.23 vs females 10.00± 3.61cm^3^, *p*≤ 0.01). In addition, this significant difference in SSV between the two genders was observed in studies conducted by Pirinc *et al*. [ 35 ] and Lentzen *et al*. [ 28 ] as well as our study that concluded males have a statistically significant larger SSV than females (*p*< 0.05). Nevertheless, a study by Oliveira *et al*. [ 36 ] used helical CT to assess the SSV of 27 females and 20 males aged 18-86 years and observed no significant difference in the sinus volume between the two genders. This could be attributed to the small sample size compared to the wide age range used in this study.

Successful transsphenoidal endoscopic surgery requires a detailed preoperative morphological evaluation of the sphenoid sinus. This is because the sphenoid sinus is surrounded by vital structures such as the internal carotid artery, optic nerve, and maxillary nerve [ 13 - 15 ]. Knowledge of the shape and pneumatization patterns of the sphenoid sinus can help assess the risk of accidentally damaging this area [ 11 ]. From a forensic point of view, if the morphology of the sphenoid sinus and gender are related, the finding could be useful in distinguishing between the genders. The morphology of the sphenoid sinus was categorized into four types in the present study. In our study, the most common morphology was the sellar type (50.5%), followed by the postsellar (40.3%) and presellar (9.2%) types. The prevalence of different types of sphenoid sinus morphology varies in different studies; however, the most common morphology is the sellar type [ 11 , 14 , 35 ], which is consistent with the present study. The absence of conchal type morphology in our study was similar to the findings of Hiremath *et al*. [ 37 ]. However, in their study, in contrast to our results, postsellar morphology was the most common type (76.6%). In addition, the results of the current study found no significant statistical difference in the prevalence of different sphenoid sinus morphologies between both genders (*p*= 0.63). Thus, sphenoid sinus morphology was not a suitable variable for gender discrimination.

The DFA was used for sex determination based on SSV in the present study. The independent variable in the present study was SSV, and the grouping variable was the gender of the understudied population. The results of the current study showed that the accuracy of sex determination was 86.0% and 92.9% in males and females, respectively. In simple terms, using the SSV, 92.9% of the females were determined as female and diagnostic accuracy was 86.0% for males; 14.0% of them were wrongly categorized as female. The efficiency of paranasal sinuses in sex determination using DFA has been reported in various studies [ 8 - 9 , 24 , 38 - 40 ]. The ability to differentiate gender by maxillary sinuses has been reported in different studies, such as Uthman *et al*. [ 6 ] (73.9%), Amin *et al*. [ 38 ] (66.7%), Radulesco *et al*. [ 39 ] (68.0%), and Paknahad *et al*. [ 24 ] (76.0%). These measurements varied from 66.7% to 76.0%. The sex determination strength of frontal sinuses in the studies of Uthman *et al*. [ 8 ] and Michel *et al*. [ 40 ] was 76.9% and 72.5%, respectively. Compared to our study, frontal and maxillary sinuses alone had a lower sex determination capability. However, Wanzeler *et al*. [ 9 ], who used maxillary, frontal, and sphenoid sinuses and foramen magnum dimensions in sex determination, concluded that when all the three sinuses were used in sex determination, the diagnostic accuracy was 96.2% in males and 92.7% in females, and if the foramen magnum dimensions were added to the equations, the accuracy increased to 100%. Furthermore, in their study, the effectiveness of SSV in sex determination between males and females was 73.7% and 81.93%, respectively. The differences between the reported accuracy in the various studies could be attributed to race, statistical, radiographic techniques, and sample size differences. The difference in race has been reported as an influencing factor in the difference between the volumes of paranasal sinuses, which could explain the dissimilarities in previous studies [ 41 ].

This study had some limitations. The study was conducted in Mashhad, which is the second most populated city of Iran, located in the northeast of the country. All the selected samples were only from the North East of Iran, and therefore do not reflect the total Iranian population. Another limitation was that all the images were evaluated by only one examiner. We recommend that further studies be conducted in different geographical areas of Iran with larger samples so that the results can be generalized to the entire Iranian population.

## Conclusion

Based on the results of this study, although SSV has been a reliable variable in sex determination in a northeastern Iranian population, the morphology of the sphenoid sinus is not a suitable indicator for sex determination since the morphology of the sphenoid sinus and sex are independent. To generalize these results to the entire Iranian population, multicenter national studies covering different geographical areas are required.

## Acknowledgments

Due to the retrospective nature of this study, which focused on archived images, the CBCT images were studied anonymously. For this reason, patient identities remained unknown. This research did not receive any specific grant from funding agencies in the public, commercial, or not-for-profit sectors.

## Conflict of Interest

The authors declare that they have no conflict of interest.
